# Mechanical Properties of Reactive Powder Concrete with Coal Gangue as Sand Replacement

**DOI:** 10.3390/ma15051807

**Published:** 2022-02-28

**Authors:** Wen Luo, Haijun Wang, Xiongwei Li, Xiaolong Wang, Zhang Wu, Yuan Zhang, Xiaoqing Lian, Xiaojun Li

**Affiliations:** 1 CHN Energy Shendong Coal Group Co., Ltd., Shenmu 719315, China; 13947726305@139.com (W.L.); 20017147@chnenergy.com.cn (X.W.); 2 Xi’an Research Institute, China Coal Technology and Engineering Group Corp, Xi’an 710077, China; wanghaijun@cctegitc.com (H.W.); lixiongwei@cctegxian.com (X.L.); wuzhang@cctegxian.com (Z.W.); 3 Graduate School, Xi’an University of Science and Technology, Xi’an 710054, China; zhangyuanwsbn@163.com; 4 College of Materials Science and Engineering, Xi’an University of Science and Technology, Xi’an 710054, China; lianxiaoqing@xust.edu.cn; 5 College of Safety Science and Engineering, Xi’an University of Science and Technology, Xi’an 710054, China

**Keywords:** coal gangue, reactive powder concrete, aggregates, back shrinkage of strength

## Abstract

Coal gangue (CG) represents a huge amount of industrial solid waste in China, and usually is used as a coarse aggregate to produce low-strength coal-gangue-based concrete. In this paper, in order to prove the possibility to obtain a higher-strength concrete with a higher CG utilization rate, reactive powder concrete (RPC) with coal gangue as a sand replacement at different replacement ratios was studied. RPC samples were prepared by replacing natural river sand (RS) with CG sand at different CG/RS weight ratios from 0–100% at intervals of 25%. Mechanical tests were carried out, and the microstructure features of RPC samples at 28 days were characterized. The test results showed that strong back shrinkage of strength existed. On days 7 and 14, the flexural strengths of samples with CG/RS replacement ratios of 0–75% fluctuated around the mean value. Strengths of samples with a CG/RS replacement ratio of 100% dropped off. However, on day 28, the flexural strengths of samples with CG were all lower than the strengths of samples on days 7 and 14. The flexural strengths and compressive strengths of the RPC with a CG/RS replacement ratio of 100% on day 14 were 14.09 MPa and 37.03 MPa, respectively, which decreased to 6.42 MPa and 28.44 MPa, respectively, on day 28. Compared with natural river sand, CG sand reduced the working performance, compressive strength, and flexural strength of RPC. Microscopic analysis showed that on day 28, increasing the CG replacement ratio could inhibit cement hydration, weaken the interface transition zone, and lead to the degradation of the RPC’s performance. Modification of CG sand would be helpful to obtain higher-strength concrete.

## 1. Introduction

Coal gangue (CG) is the waste produced during coal mining and coal preparation plant operations, and is the largest source of industrial solid waste in China [1]. Accumulation of CG causes serious problems, such as the reduction in arable land, emissions of toxic and harmful gases due to its spontaneous combustion, and leaching of toxic and harmful elements into underground water and soil [2]. Efficient and environmentally friendly recycling of CG is beneficial to the Chinese economy and global environment protection [3]. Coal gangue (CG) is usually used as a coarse aggregate to produce low-strength coal-gangue-based concrete [4,5]. The fundamental links between the microscopic properties of CG and the macroscopic properties of concrete are not clear yet [6].

Reactive powder concrete (RPC) is a type of ultrahigh-performance concrete developed through microstructure reinforcement technology [7]. It generally has better mechanical properties and durability than traditional concrete. Its excellent performance is attributed to the use of microstructural engineering methods, such as removal of coarse aggregates, use of a low water–binder ratio, reduction in the ratio of CaO to SiO_2_, and addition of steel microfibers [8]. Because CG is rich in silica and its material composition is similar to sand, using CG sand in RPC would be a potential approach to reuse CG at a large scale [9,10].

This study mainly investigated the potential reuse of CG sand in RPC. In order to prove the possibility to obtain a higher-strength concrete with a higher CG utilization rate, reactive powder concrete (RPC) with coal gangue as a sand replacement at different mix ratios was studied.

The formability and indexes of RPC, including water absorption, porosity, flowability, and flexural and compressive strength, were tested. Furthermore, the rationality of the RPC ratio was evaluated by scanning electron microscopy (SEM) and X-ray diffraction (XRD). The phase composition and interfacial transition zone (ITZ) around the aggregate in the RPC were observed to reveal the mechanism underlying the change in the mechanical properties of RPC with the addition of CG. The results of this study are expected to contribute to the recycling of CG and to the improvement of the sustainability of coal resources.

## 2. Materials and Methods

### 2.1. Raw Materials

#### 2.1.1. Binders and Fibers

**Figure 1:**
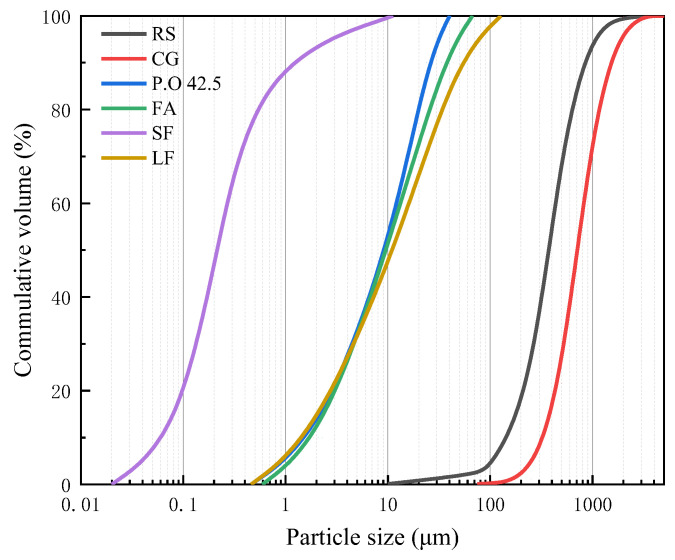
Particle size distribution of the raw materials.

Ordinary Portland Cement P•O 42.5, produced under the Chinese standard GB175-2007, was used in the RPC. Fly ash (FA), silica fume (SF), and limestone flour (LF) were added as supplementary cementitious materials. The mean particle sizes (D_50_) of the P•O 42.5, FA, SF, and LF as obtained by a laser particle size analyzer (MAZ 3000, Malvern-PANalyticalMalvern, England) were 10.3, 9.9, 0.2, and 12.0 μm, respectively. Further, straight copper-coated steel fibers that were 10 mm in length and 0.02 mm in diameter and with a density of 7850 kg/m^3^ and a tensile strength greater than 3000 MPa were added to the cement mixture to increase the flexural and tensile strengths. A polycarboxylic ether superplasticizer (SP) with 55% solid content was added to this mixture. The chemical composition of the mixture powder was tested by X-ray fluorescence (XRF, S2 PICOFOX, Bruker, Karlsruhe, Germany).and is listed in Table 1. The size distribution of each component of the mixture is shown in Figure 1 [11,12].

#### 2.1.2. Fine Aggregates

Local natural river sand (RS) and crushed CG were used as fine aggregates. The CG was collected from a waste disposal site in Budiertai, Inner Mongolia, for substituting parts of the RS as the aggregate. Both the RS and CG were crushed to a maximum particle size of 2 mm, and their size distribution results are shown in Figure 1. The crushed CG had a small density, high porosity, and large specific surface area [4]. The chemical compositions of the two materials determined using X-ray fluorescence (XRF) are listed in Table 2.

**Figure 2:**
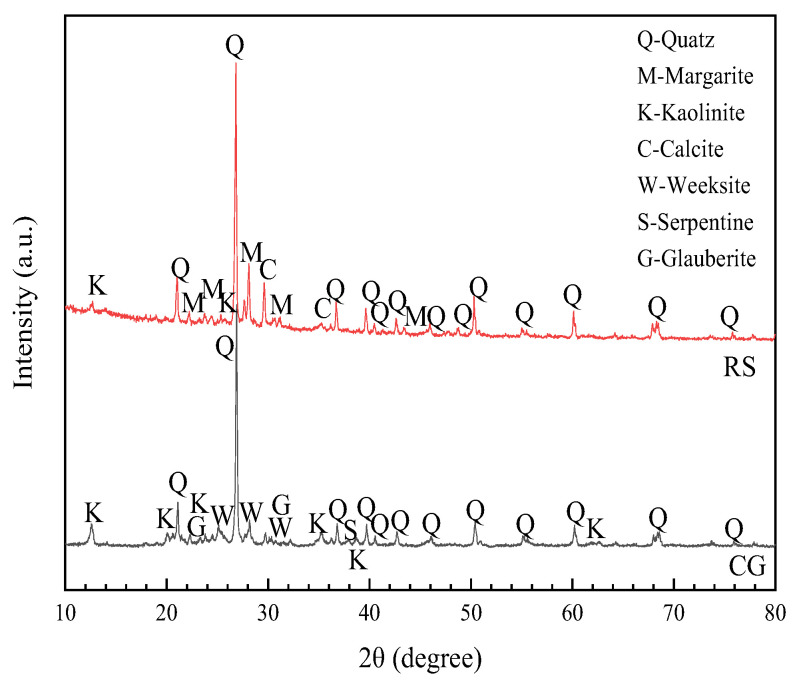
XRD patterns of fine aggregates.

The XRD (XRD-7000, Shimadzu, Kyoto, Japan) test results for the RS and CG are shown in Figure 2. The RS was mainly composed of quartz, margarite, kaolinite, and calcite. The material composition of the CG was similar to that of the RS, with both having quartz as the main component. Compared with the RS, the main material composition of the CG contained a large amount of kaolinite, which is a characteristic component of CG. Kaolinite is rich in Al_2_O_3_, so CG provides a certain amount of active component (Al_2_O_3_) for cement mortar, which can be hydrated with cement to produce ettringite.

**Figure 3:**
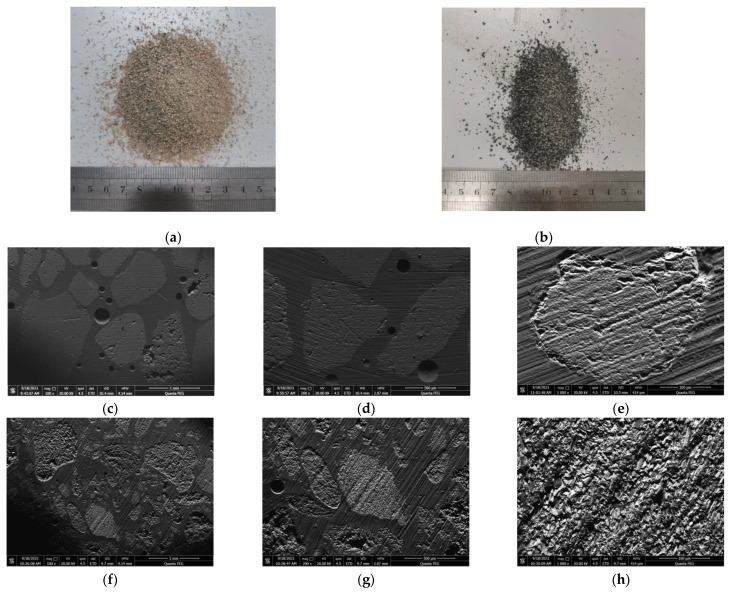
Macromorphology and microstructure of fine aggregates: (**a**) macromorphology of RS; (**b**) macromorphology of CG; (**c**–**e**) SEM images of RS; (**f**–**h**) SEM images of CG.

The microstructures of the CG and RS were characterized by SEM (Quanta FEI 250, FEI, Hillsboro, NJ, USA), and the results are shown in Figure 3. Clearly, the RS was smooth and clean with mostly a polygonal shape, whereas the morphology of the CG was more complex. Compared with the RS, the CG had a larger specific surface area, more irregular boundary contours, and a more complex shape. In addition, unlike that of the RS, the section of the CG had more needlelike protrusions and developed microscopic pores. The basic physical properties of the fine aggregates are listed in Table 3. The CG had a smaller apparent density and bulk density, as well as a higher porosity, which was consistent with its porous microstructure shown in Figure 3. The working performance and strength of the CG was estimated to be lower than that of the RS.

### 2.2. Mix Proportions

#### 2.2.1. Mixture with Different CG/RS Ratios

The mix proportions designed in this study are listed in Table 4. The CG0 mixture was prepared using RS as the fine aggregate, and four mixtures, namely CG25, CG50, CG75, and CG100, were prepared by substituting RS with CG at ratios of 25%, 50%, 75%, and 100%, respectively. The replacement ratio for the CG was calculated by weight fraction. Cement content was set as 37.8%. To prepare more environmentally friendly concrete, the proportion of P•O 42.5 was lowered and that of FA was increased. Steel fibers were added to the mixture at 160 kg/m^3^, and water and liquid admixtures were mixed at a ratio of 1:6.7. To utilize the CG as much as possible, the ratio of aggregate to binder material was 1.65.

#### 2.2.2. Mixture with Different Fiber Content

The tensile strength of RPC is mainly determined by the added fibers, which also affect the compressive strength and fluidity of concrete to a certain extent. Hence, the role of steel fibers in the performance and strength of RPC with CG was experimentally evaluated. In this experiment, the steel fiber content in the proportioning scheme shown in Table 4 was reduced to 80 and 0 kg separately, and the fluidity and strength (on day 28) of each RPC case were compared to determine the role of steel fibers in each proportioning scheme.

### 2.3. Specimen Preparation

Mixtures were prepared using a mortar mixer using a well-established procedure for RPC according to previous studies [13,14]. First, all the dry mixtures, including RS, CG, P•O 42.5, FA, SF, and LF, were stirred for 3 min at a low speed of 60 rpm. Then, the mixtures were mixed continuously at a high speed of 200 rpm for 7 min (the mixing time was adjusted according to the actual situation), while gradually adding a 75% solution of SP and water. The remaining 25% was added dropwise when the fluidity of the mixture nearly approached the desired value. Finally, steel fibers were added slowly and stirred at a speed of 60 rpm until homogenous mixtures were obtained.

Fresh mixtures were poured into plastic molds with dimensions of 40 mm × 40 mm × 160 mm and then vibrated for 1 min to remove bubbles formed in the mixing process. Then, the molds were covered with plastic sheets to avoid surface moisture loss by the specimens, and were stored in natural conditions in a curing room at 20 ± 1 °C and 95% relative humidity (RH) for 48 h. After demolding, the specimens were stream-cured at 95 ± 1 °C and 95% RH for 24 h. Finally, the specimens continued to be naturally stored at 20 ± 1 °C and 95% RH until the testing times, namely 7, 14, and 28 days.

### 2.4. Test Methods

#### 2.4.1. Packing Density

The modified Andreasen and Andersen model [15] was employed to evaluate the packing density of dry mixtures as follows:

(1) $$P\left(D\right)=\frac{D^{q}-D_{min}^{q}}{D_{max}^{q}-D_{min}^{q}}$$

where *D* represents the particle size of raw materials with a unit of μm; P(*D*) is the accumulation fraction of particles finer than size *D*; *D_min_* and *D_max_* are the minimum and maximum particle sizes in the mixtures, respectively; and *q* is the distribution modulus, which was set as 0.26 in this study.

#### 2.4.2. Fluidity Test

Fluidity tests were conducted in accordance with the American Society for Testing and Materials (ASTM) C230/C230-14 to evaluate the workability of the fresh mixtures. Note that the fresh mixtures to be tested included a cement slurry containing different proportions of steel fibers.

#### 2.4.3. Mechanical Test and Water Absorption

The flexural and compressive strengths on days 7, 14, and 28 were measured using a TYE-300 universal testing machine with a loading rate of 2 mm/min. The flexural strength was tested using a three-point method with a span distance of 100 mm. The mean value of the results of the three specimens was reported as the flexural strength for each mixture. Then, the compressive-strength test was conducted on the three broken specimens. The average of three results was considered the compressive strength for each mixture.

The water absorption and porosity of the specimens on day 28 were measured in accordance with ASTM C 1585-2013. Three parallel samples with dimensions of 40 mm × 40 mm × 60 mm were cut after the mechanical tests, and their mean values were obtained as results.

#### 2.4.4. SEM and XRD

The concrete samples with different CG substitution rates were characterized through XRD at a scan rate of 5° (2θ)/min from 5° to 80°. The hydration reaction was studied on days 7 and 28 under RPC with different CG substitution rates. Scanning electron microscopy (SEM) was used to analyze the microstructures of the concrete specimens with different CG replacement rates. The morphology of hydration products and microstructure of the interfacial transition zone (ITZ) were analyzed by observing the microscopic images of the RPC surface and section and combining these results with XRD information.

## 3. Results and Discussion

### 3.1. Mixture with Different CG/RG Ratios

#### 3.1.1. Packing Density

**Figure 4:**
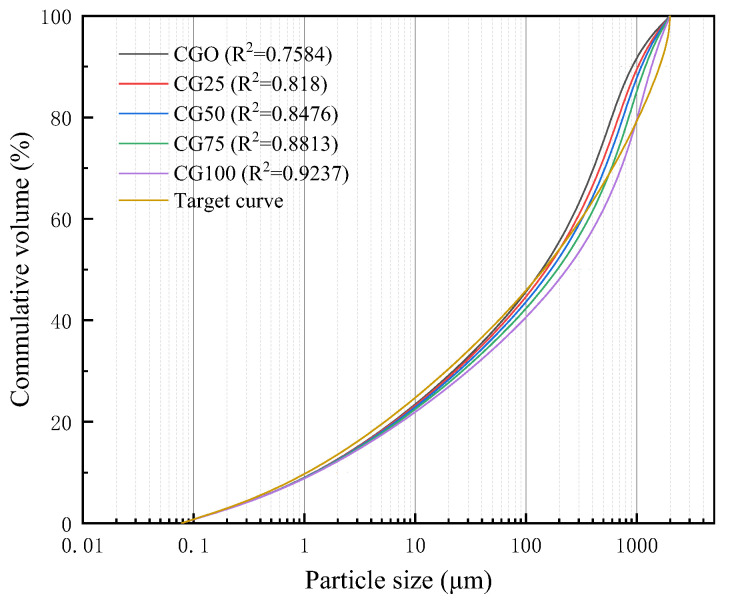
Particle size distribution of combined dry mixtures. R^2^ = coefficient of determination.

The particle size distribution of dry mixtures with CG at different replacement ratios is shown in Figure 4. The target curve was calculated according to Equation (1), in which the minimum particle size of the SF, *D_min_*, was 0.0215 μm; and the maximum particle size of the RS and CG, *D_max_*, was 2000 μm. The curves of the other five dry mixtures were obtained by sieving the dry mixtures to the required particle size. A high coefficient of determination (R^2^) was related to a high packing density of the dry mixtures. The coefficients of determination for CG0, CG25, CG50, CG75, and CG100 were 0.7584, 0.818, 0.8476, 0.8813, and 0.9237, respectively, and increased gradually with increasing replacement ratios of CG, achieving the maximum value at a 100% replacement ratio.

These results indicated that the inclusion of CG increased the packing density of the dry mixtures. According to the particle size distribution of the RS and CG shown in Figure 1, the particle size of the RS was concentrated in the range of 0.25–0.50 mm, while that of the CG was concentrated in the range of 1.00–0.50 mm. Due to the poor performance of the CG as an aggregate, particles with a large size (0.25–2 mm) were deliberately selected after screening to improve its working performance; therefore, large R^2^ values were shown for CG0 to CG100. However, because natural RS has a small particle size, particles with a large particle size lacked in the system, resulting in the maximum R^2^ for CG100.

#### 3.1.2. Fluidity

**Figure 5:**
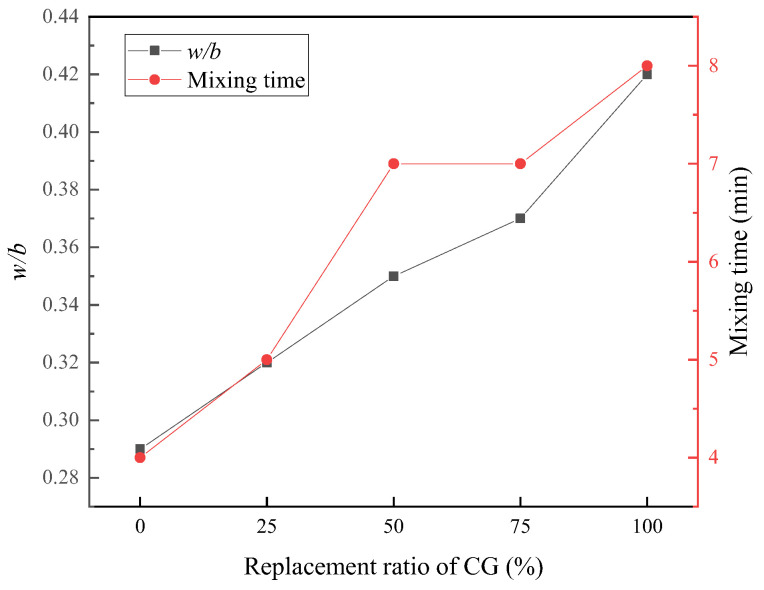
Variation of w/b and mixing time with the replacement ratio of CG when the fluidity was controlled at 200 mm.

The fluidity of the RPC varied greatly with the change in CG replacement ratios. In this experiment, the fluidity of five specimens of RPC was uniformly controlled at 200 mm (±10 mm). The variation in w/b with the CG replacement ratio is shown in Figure 5. The figure shows that when the fluidity was 200 mm (±10 mm), the w/b increased continuously with increasing CG replacement ratios, in the range of 0.29–0.42. Similarly, the mixing time directly reflected the performance of the cement slurry with each CG replacement ratio. As the figure shows, with increasing CG content, the stirring time changed from 4 min to 8 min. Because the CG was loose and porous, it first absorbed some of the water and water-reducing agent used for the hydration reaction.

Compared with ordinary crushed stone, CG aggregate has a loose structure and comprises more flaky particles and pores; moreover, its properties can fluctuate considerably. Although CG has an apparent density similar to that of natural gravel, it is porous, and has a higher water-absorption ability. Owing to the large porosity of CG aggregate and a high number of microcracks in the crushing process, CG aggregate generally has a higher water absorption than natural gravel, and will absorb water in the fresh concrete during the mixing process, which seriously affects the working performance of concrete with CG aggregates [16,17].

#### 3.1.3. Mechanical Test and Water Absorption

**Figure 6:**
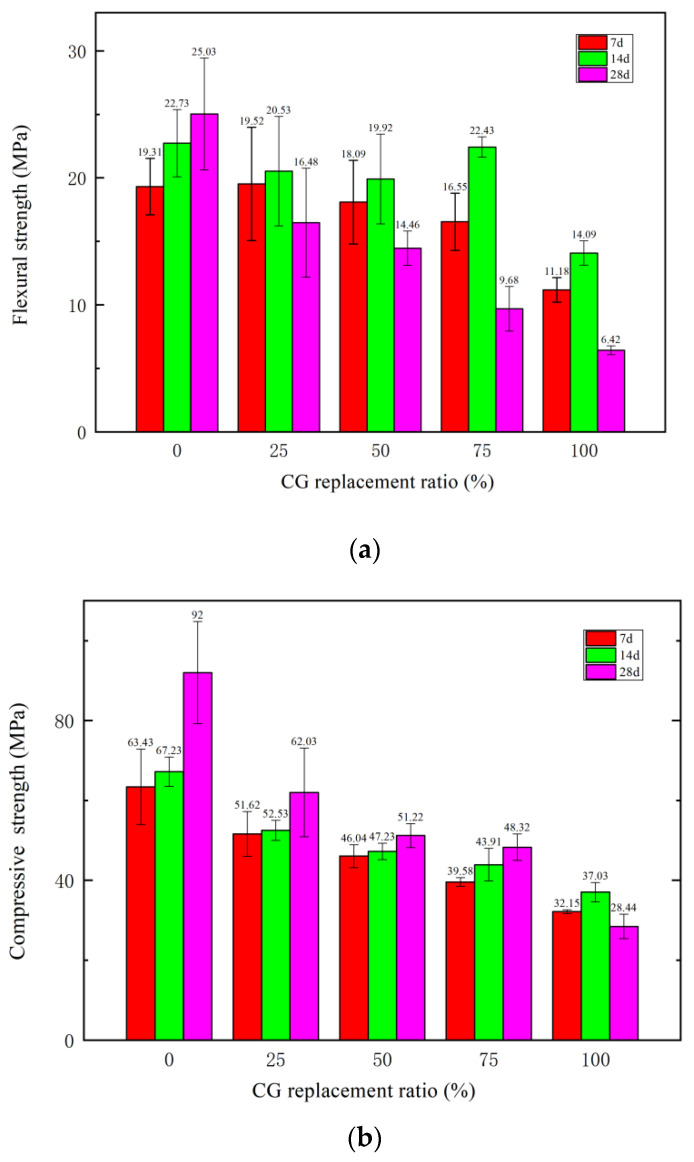
Mechanical strength of hardened specimens with different CG replacement ratios on days 7, 14, and 28: (**a**) flexural strength; (**b**) compressive strength.

The flexural and compressive strengths of the specimens with CG at different replacement ratios on days 7, 14, and 28 are shown in Figure 6.

The flexural strength was mainly determined by the cementation strength of the concrete, the tensile strength of the steel fiber, the distribution of the steel fiber, and the wrapping tightness of the cementation material and steel fiber. As shown in Figure 6, with an increasing CG replacement ratio, the flexural strength of the concrete showed an overall decreasing trend. On day 7, the flexural strengths of samples with CG/RS replacement ratios of 0–75% fluctuated from 16.55 to 19.52 MPa, and dropped to 11.18 MPa with a replacement ratio of 100%. On day 14, the flexural strengths of samples with CG/RS replacement of 0–75% fluctuated from 19.92 to 22.73 MPa, and dropped to 14.09 MPa with a replacement ratio of 100%. On day 28, the flexural strengths of samples with CG were all lower than the strengths of samples on days 7 and 14. The test results showed strong back shrinkage of the strength, which was caused by the degradation of the structure of the hardened cement paste due to appearance, broadening, and connection of microcracks in hardening structure.

With the increasing CG replacement ratio, the compressive strength of the concrete decreased gradually. The compressive strengths of CG0 to CG100 on day 14 were 67.23, 52.53, 47.23, 43.91, and 37.03 MPa, respectively. This was because the strength of the CG aggregate was low, and the hydration reaction of the concrete was degraded with the addition of CG, resulting in a low density, high porosity, and loose structure of the concrete due to the CG aggregate. The compressive strength of the specimens generally increased from day 7 to day 28, except with a CG replacement ratio of 100%. Back shrinkage of the compressive strength occurred when the CG replacement ratio was 100%.

This was due to a crystal form change in the later stage of hydration, in which a low water-to-binder (w/b) ratio causes some microcracks in the hardened cement paste due to its volume expansion, resulting in a degradation in the properties of the RPC matrix [18,19]. In addition, the appearance, broadening, and connection of microcracks in the hardened cement paste caused more flexural strength loss than compressive strength loss [20].

**Figure 7:**
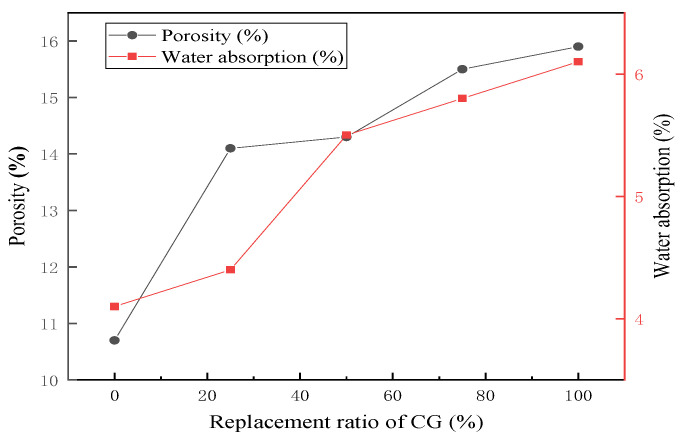
Water absorption and porosity of specimens with different CG replacement ratios.

Water absorption and porosity were directly related to the durability of the RPC. Figure 7 shows the water absorption and porosity of specimens with different CG replacement ratios on day 28. Water absorption and porosity of specimens increased with increasing CG replacement ratios. The water absorption and porosity of CG100 were 15.9% and 6.1%, respectively, which were two or three times as much as those of CG0. The pore structure of the CG and a large w/b were the main reasons for the increases in water absorption and porosity [21].

**Figure 8:**
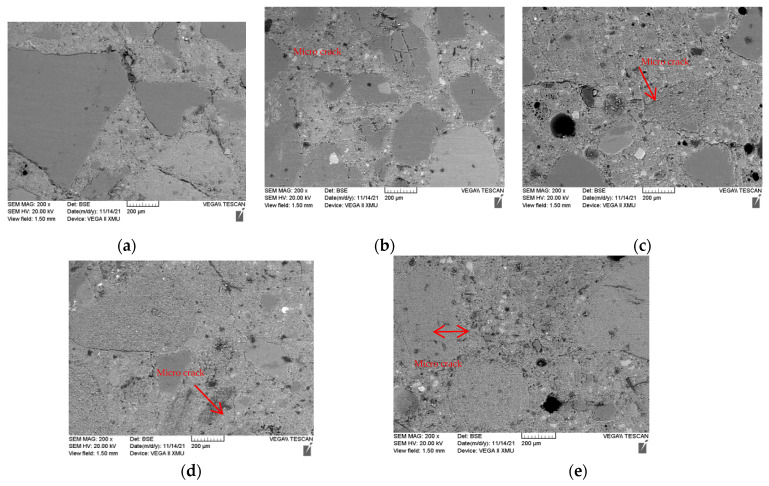
BSE images of specimens on day 28: (**a**) CG0; (**b**) CG25; (**c**) CG50; (**d**) CG75; (**e**) CG100.

Figure 8 shows the back-scattering electron microscope (BSE) images of the microstructures of specimens with different CG replacement ratios on day 28. The BSE images mainly reflect the distribution of elements on the specimen surface. In the figure, RS is indicated by the grey-black region with a dense and smooth section, whereas the region where CG was located is bright, and shows a continuous uniform and loose multispace morphology. The darkest areas are pores or impurities. Thus, the RS and CG can be clearly distinguished from each other. Clearly, as the CG replacement ratio increased, the number of loose and porous particles increased. Fewer pores of different shapes and sizes can be observed in the images of CG0 specimens; however, they became more pronounced with increasing CG replacement ratios. The large holes may be filler holes or residual bubbles. As the CG replacement ratio increased, more large spherical holes and a large number of pores were observed due to the CG. A large number of white Ca(OH)_2_ hydration products were found in the cement matrix. However, as the CG replacement ratio increased, the amount of the substance in the white region decreased. In certain cases, the hydration reaction became less sufficient, and the cement matrix became looser with the increasing CG replacement ratio.

### 3.2. Mixes with Different Fiber Ratios

To explore the effects of steel fibers on the concrete’s performance and mechanical properties, and for the CG aggregate tests, the content of steel fibers was decreased from 160 to 80 kg/m^3^ and 0 kg/m^3^, respectively, for the above-mentioned CG replacement rates; then, the changes in fluidity and strength (on day 28) were compared.

**Figure 9:**
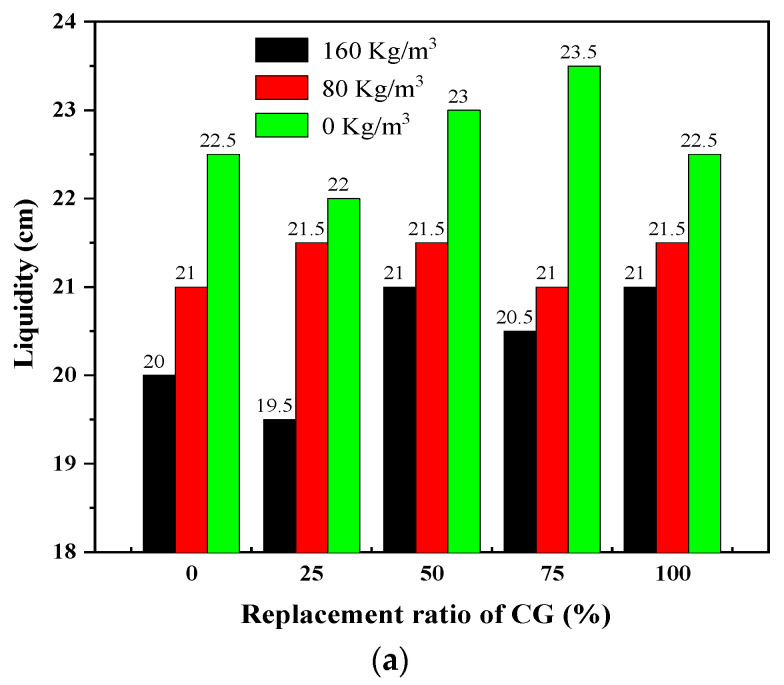

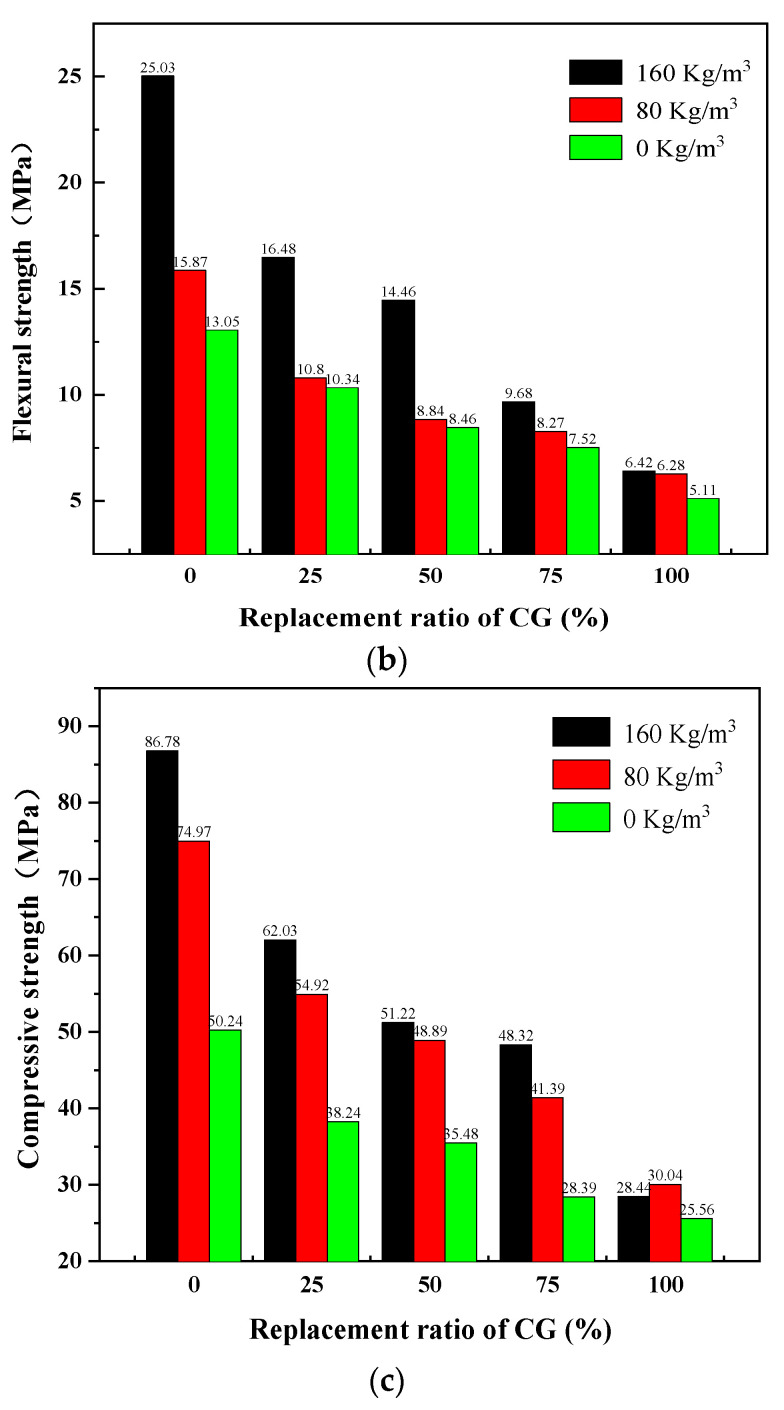
Mechanical strength of hardened specimens with different CG replacement ratios on days 3, 7, 28, and 60: (**a**) fluidity; (**b**) flexural strength; (**c**) compressive strength.

The content of steel fibers considerably affected the fluidity of the RPC. With the increase in fiber content, the fluidity of the concrete decreased significantly. As shown in Figure 9, the fluidity of the cement mortar with different CG replacement ratios greatly improved when the content of steel fibers was reduced. When the steel fiber content was lowered from 160 to 0 kg/m^3^, the fluidity increased by 1.5–2.5 cm. This was mainly because the steel fibers served as a skeleton when they were mixed into the matrix, which prevented the flow of concrete. In addition, the steel fibers absorbed part of the water in the matrix, reducing the fluidity of the concrete.

In terms of flexural strength, as shown in Figure 9, as the steel fiber content decreased, the flexural strength of the concrete with different CG replacement ratios decreased. When the steel fiber content was lowered from 160 to 0 kg/m^3^, the flexural strength dropped to a range of 1.31–11.98 MPa. Specifically, with the increase in the CG replacement ratio, the effects of steel fibers on the flexural strength of the concrete decreased. This was because the factors of the steel fibers that contributed to the improvement in the flexural strength of the concrete were mainly determined by the strength of the steel fibers themselves and the degree of combination between the steel fibers and the concrete matrix. As the CG replacement ratio increased, the process of the hydration reaction was restrained or slowed down, leading to the reduction in hydration products and weakening of the combination degree between the steel fibers and the matrix. Therefore, the increase or decrease in the steel fiber content had little effect on the concrete with a high CG substitution rate.

In terms of compressive strength, as shown in Figure 9, with the decrease in the steel fiber content, the compressive strength of the concrete at each CG replacement ratio showed a decreasing trend. When the steel fiber content was lowered from 160 kg/m^3^ to 0 kg/m^3^, the compressive strength decreased to a range of 2.88–36.54 MPa. This was because the addition of steel fibers increases the ductility of concrete, reduced the phenomenon of stress concentration in the concrete, and made the stress in the concrete more uniform in the test process. Moreover, the steel fibers serves as a skeleton in the matrix to compensate for the lack of aggregate strength [22].

### 3.3. Hydration

**Figure 10:**
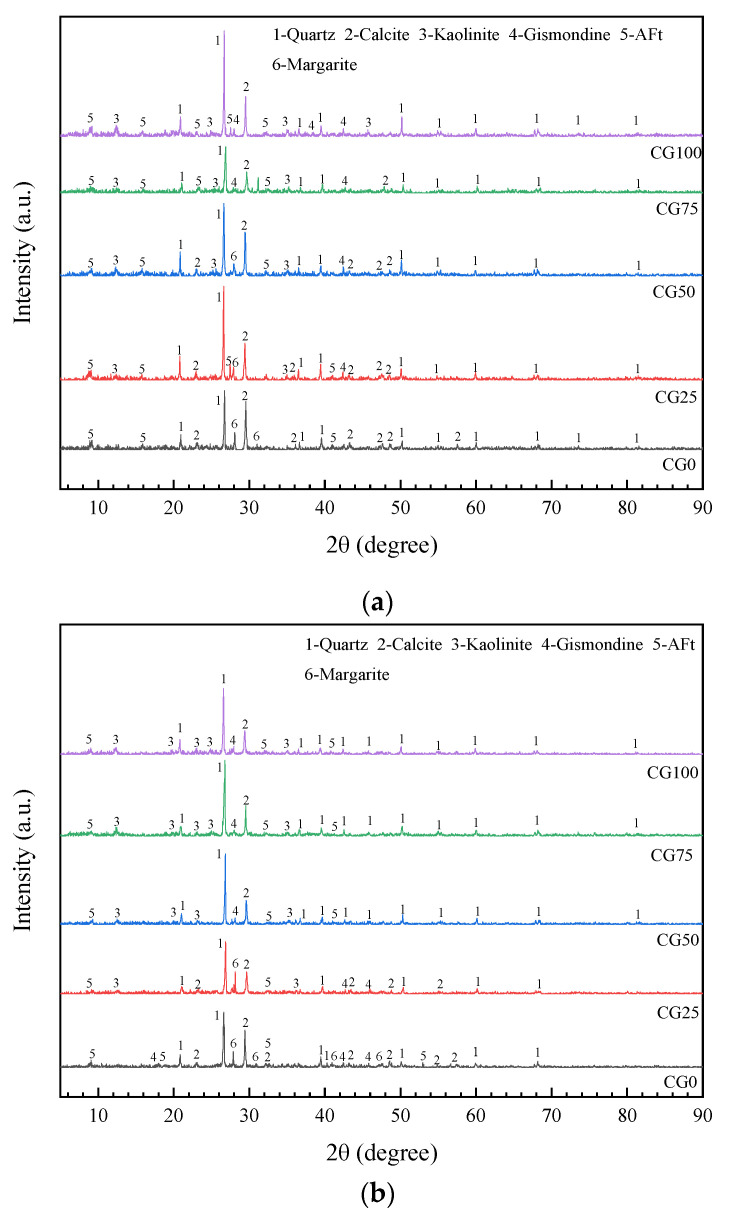
XRD patterns of specimens with different CG replacement ratios: (**a**) day 7; (**b**) day 28.

**Figure 11:**
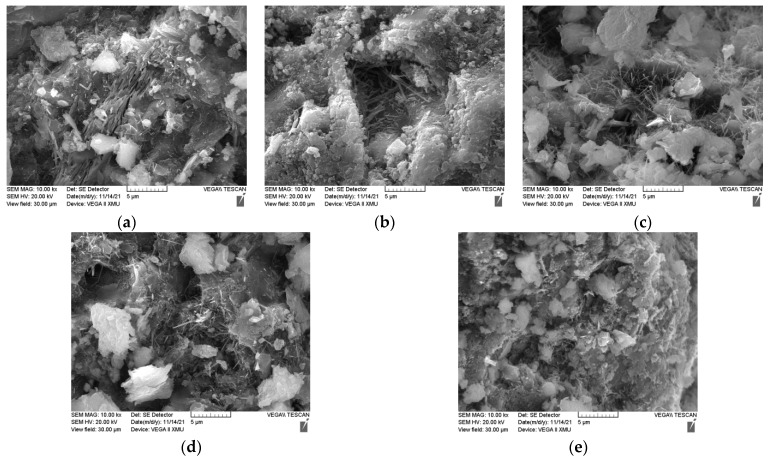
Hydration reaction products with different CG replacement ratios on day 28: (**a**) CG0; (**b**) CG25; (**c**) CG50; (**d**) GC75; (**e**) CG100.

Figure 10 shows the XRD patterns of the concrete on days 7 and 28 with different CG replacement ratios; Figure 11 shows the microscopic morphology of hydration products of concrete on day 28 under the condition of different CG replacement ratios.

A comparative analysis of the XRD images indicated that diffraction products mainly fell into two categories. One was raw materials, including quartz, margarite, kaolinite, and calcite. Quartz and margarite were the main components of the river sand, and quartz and kaolinite were the main components of the CG sand. Calcite was the main component of the LF. The other was hydration products, including gismondine, AFt, and C-H-S. In the XRD patterns, with an increase in the CG ratio, the peak for margarite gradually weakened and the peak for kaolin was gradually enhanced, which was closely related to the phase compositions of the RS and CG, respectively. The XRD patterns for days 7 and 28 show similar variation trends. In terms of hydration products from day 7 to day 28, C-H-S existed mostly in the form of a gel in the concrete, and no crystallization peak was observed. With the increase in the CG replacement ratio, gismondine appeared increasingly frequently, because the reaction of active Al_2_O_3_ and SiO_2_ in the CG with the cement easily generated gismondine. The hydration product of CG0 was mainly AFt. On day 28, the hydration products were more abundant, the number of different hydration products was greater, and the diffraction was more obvious, indicating that the hydration reaction occurred effectively.

In the CG100, hydration products on days 7 and 28 had hardly changed, because the hydration reaction in the early stage consumed a large amount of water, resulting in insufficient hydration reaction in the later stage. In terms of the microstructure of hydration products, as shown in Figure 11, for CG0, a large amount of hydration products, such as fibrous C-H-S and needlelike and columnar Aft, were crosslinked and filled in the pores, thereby forming a skeleton network system. With the increase in the CG replacement ratio, the skeleton network system became sparse, and a relatively lower amount of the needlelike material appeared [23,24]. From day 7 to day 28, the diffraction peaks for quartz at all angles under the same CG replacement ratio were significantly enhanced, and the peak width in the diffraction pattern of the mortar was decreased, because the new quartz crystal had a smaller width than the original, which was conducive to the increase in the strength of mortar. The diffraction peak for kaolinite in the CG had no significant change in quantity in the diffraction pattern of the mortar at each CG replacement ratio; however, the diffraction intensity was slightly increased, which indicated that kaolinite participated in the hydration reaction to generate partially hydrated kaolin, which could be the cause of the back shrinkage of the strength of the samples.

### 3.4. Microstructure

**Figure 12:**
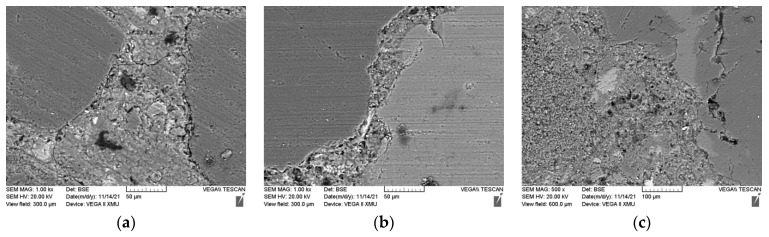

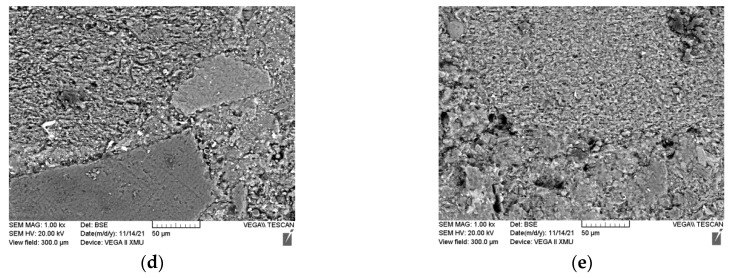
ITZ microstructure of specimens with different CG replacement ratios on day 28: (**a**) CG0; (**b**) CG25; (**c**) CG50; (**d**) GC75; (**e**) CG1000.

The ITZ was the weakest structural region in the concrete. Figure 12 shows the ITZ around the RS and CG of the CG0, CG50, and CG100 on day 28. When the fine aggregate was RS, its polygonal shape helped it to be tightly wrapped by the cement matrix, with almost no cracks around it, and the cement matrix was relatively compact. With the increase in the CG replacement ratio, the cement matrix was still tightly wrapped the aggregate, with no obvious change and almost no cracks around it. However, when the aggregate was CG, the cement matrix became significantly looser, and the aggregate wrapped by the cement matrix was less dense. Cracks could be clearly seen, and the interface was not complete and was broken. This affected the strength from a macro perspective. As the CG replacement rate increased, owing to the low density of the CG, the surface area of the CG increased and part of the CG was exposed, resulting in a sharp decrease in the mechanical strength. The hydration product Ca(HO)_2_ in the white area and the bubbles in the black area in the figure can be observed: with the increase in the CG replacement rate, the number of hydration products and bubbles in the CG100 could be obviously observed to decrease. This showed that the cement matrix was relatively loose, and the CG inhibited the hydration reaction, causing ring breakage in the interface structure to a certain extent [25,26].

## 4. Conclusions

This study investigated the application of CG in RPC as a substitute for RS. The flow and mechanical properties of the RPC varied with the steel fiber content and CG substitution rate. The effects of the replacement ratio of CG on the hydration behavior and the structure in the interfacial transition zone were analyzed by XRD, SEM, and ESD. The main results were as follows:

1. On days 7 and 14, the flexural strengths of samples with CG/RS weight ratios of 0–75% fluctuated around the mean value. Strengths of samples with CG/RS weight ratios of 100% dropped off. On day 28, the flexural strengths of samples containing CG were all lower than the strengths of samples on days 7 and 14. The flexural strengths of the RPC with a CG/RS replacement ratio of 100% on days 7 and day 14 were 11.18 MPa and 14.09 MPa, respectively, and decreased to 6.42 MPa on day 28.
2. The compressive strength of the RPC with a CG/RS ratio of 100% on day 14 was 37.03 MPa, meeting the design requirements of C35 strength grade concrete, but on day 28, the compressive strength had decreased to 28.44 MPa.
3. Strong back shrinkage of strength existed. A low water-to-binder (W/B) ratio caused some microcracks in the hardened cement paste due to its volume expansion, resulting in a degradation in the properties of the RPC matrix. More flexural strength loss than compressive strength loss occurred. Kaolinite participated in the hydration reaction to generate partially hydrated kaolin, which could have been the cause of the back shrinkage of strength.
4. Steel fibers greatly improved the compressive strength and tensile strength of the RPC; however, they also decreased the fluidity. With the increasing CG replacement ratio, the main observed hydration products were transferred from AFt to AFt and gismondine. The CG inhibited the hydration reaction in the CG100 compared with that in the CG0.
5. Compared with natural river sand, the CG sand reduced the working performance, compressive strength, and flexural strength of the RPC. A microscopic analysis showed that on day 28, increasing the CG ratio inhibited cement hydration, weakened the interface transition zone, and led to the degradation in RPC performance. Modification of the CG sand would be helpful to obtain higher-strength concrete.

## Figures and Tables

**Table 1:** Chemical compositions of binders (%).

Material	SiO_2_	Al_2_O_3_	Fe_2_O_3_	CaO	MgO	Na_2_O	K_2_O	TiO_2_	SO_3_	P_2_O_5_	Loss
P•O 42.5	23.04	4.5	4.78	64.81	2.02	0.31	0.22	-	0.636	-	2.34
FA	42.43	21.83	12.81	15.12	2.12	2.04	1.02	-	-	-	0.42
SF	95.19	-	0.13	-	0.80	-	-	-	-	-	2.81
LF	3.45	1.47	0.24	52.12	0.77	-	-	-	-	-	40.22

**Table 2:** Chemical compositions of fine aggregates (%).

Material	SiO_2_	Al_2_O_3_	Fe_2_O_3_	CaO	MgO	Na_2_O	K_2_O	TiO_2_	SO_3_	P_2_O_5_	Loss
RS	68.46	15.65	5.45	3.69	0.05	1.75	2.42	0.86	0.22	0.30	1.87
CG	68.32	17.42	4.43	2.31	1.25	1.50	2.50	0.84	0.31	0.16	10.79

**Table 3:** Physical properties of fine aggregates.

Material	Apparent Particle Density (kg/m^3^)	Bulk Density (kg/m^3^)	Porosity (%)
RS	2647	1577	40.4
CG	2357	1189	49.6

**Table 4:** Designed mix proportions (kg/m^3^).

Mixture	RS	CG	P•O 42.5	FA	SF	LF	Steel Fiber
CG0	1234	0	312	223	125	89	160/80/0
CG25	925	309	312	223	125	89	160/80/0
CG50	617	617	312	223	125	89	160/80/0
CG75	309	925	312	223	125	89	160/80/0
CG100	0	1234	312	223	125	89	160/80/0

## Data Availability

Not applicable.
